# The Structure of the TFIIH p34 Subunit Reveals a *Von Willebrand Factor A* Like Fold

**DOI:** 10.1371/journal.pone.0102389

**Published:** 2014-07-11

**Authors:** Dominik R. Schmitt, Jochen Kuper, Agnes Elias, Caroline Kisker

**Affiliations:** Rudolf Virchow Center for Experimental Biomedicine, Institute for Structural Biology, University of Wuerzburg, Wuerzburg, Germany; National Cerebral and Cardiovascular Center, Japan

## Abstract

RNA polymerase II dependent transcription and nucleotide excision repair are mediated by a multifaceted interplay of subunits within the general transcription factor II H (TFIIH). A better understanding of the molecular structure of TFIIH is the key to unravel the mechanism of action of this versatile protein complex within these vital cellular processes. The importance of this complex becomes further evident in the context of severe diseases like xeroderma pigmentosum, Cockayne's syndrome and trichothiodystrophy, that arise from single point mutations in TFIIH subunits. Here we describe the structure of the p34 subunit of the TFIIH complex from the eukaryotic thermophilic fungus *Chaetomium thermophilum*. The structure revealed that p34 contains a *von Willebrand Factor A* (vWA) like domain, a fold which is generally known to be involved in protein-protein interactions. Within TFIIH p34 strongly interacts with p44, a positive regulator of the helicase XPD. Putative protein-protein interfaces are analyzed and possible binding sites for the p34-p44 interaction suggested.

## Introduction

The TFIIH complex is a multi-subunit protein assembly involved in transcription and DNA repair. It consists of 10 different proteins forming two sub-complexes. The 7-subunit TFIIH core comprises the two helicases XPD and XPB, as well as p62, p52, p44, p34 and p8. In addition, MAT1, CycH and cdk7 form the Cyclin-Activating-Kinase complex (CAK), which is attached to the core via a direct interaction of XPD and presumably XPB with MAT1 [1], [2]. Recently, additional subunits like XPG and Tfb6 have been suggested [3], [4] and ancillary interacting partners were identified [5], emphasizing the complexity of TFIIH and the high level of dynamics to participate in different processes. While both the TFIIH core and the CAK sub-complex are essential for RNA Polymerase II mediated transcription, the release of CAK from TFIIH promotes the excision of DNA lesions during the nucleotide excision repair (NER) pathway [6]. Mutations in XPD, XPB and p8, affecting these processes, are known to be causative for xeroderma pigmentosum (XP), Cockayne's syndrome (CS) and trichothiodystrophy (TTD), severe diseases associated with TFIIH function [7]–[9]. In addition, mutations in XPB, p62 and p52, lead to photosensitivity and XP/TTD like phenotypes in Drosophila [10]–[12].

The core TFIIH assembly is highly conserved among eukaryotes, with only p8, p52 and p62 lacking in a few species [13]. Of all its subunits XPD and XPB exhibit the highest sequence conservation, while p62 is most divergent [13]. Within TFIIH the helicase and ATPase activities of XPD and XPB are tightly regulated by their associated partners p44 and p52, respectively [14], [15], both of which also interact with other subunits. The interaction of p52 with p8, the smallest of the core subunits, adds to the XPB stimulation and is essential for TFIIH stability and NER activity [16]. Moreover, p44 interacts with the p34 subunit and the depletion of this subunit from the complex leads to reduced DNA repair capacity [17]. Over the past two decades the intricate network of regulation within TFIIH has been a major research focus, especially regarding the XPD/p44 and XPB/p52/p8 subunits. However, only very little is known with respect to the other core subunits. Apart from its role in stimulation of the XPD helicase [15], an E3 ubiquitin ligase activity has been proposed for the p44 yeast homologue Ssl1 [18] whereas no enzymatic function is known for the p34 subunit of TFIIH, although it has been implicated in the process of mRNA-splicing, based on phylogenetic considerations [13].

Here we describe the first crystal structure of the TFIIH subunit p34 from *C. thermophilum* (ct), comprising its N-terminal domain, which is known to strongly interact with the C-terminal C4C4 RING domain of p44 [19],[20]. The structure reveals a *von Willebrand Factor A* (vWA) like fold, typical for other vWA containing proteins involved in a multitude of protein-protein interactions. Structural comparison allowed us to delineate similarities as well as differences to already known vWA domains, providing insight into the role of p34 within TFIIH. In addition, a putative binding site for p44ct could be mapped onto the p34ct vWA domain, based on its electrostatic surface potential and previous studies.

## Materials and Methods

### Protein Expression and Purification

The genes for p34ct full-length (1–429) and p34ct 1–277 were cloned into the pBADM-11 vector (EMBL) using ligase independent SLIC cloning [21]. Protein expression was carried out in *E. coli* BL21-CodonPlus (DE3) RIL cells grown in Lennox LB medium. After induction with 0.05% L−(+)−arabinose at an OD_600_ of 0.8 the cells were further grown for 18 – 20 hours at 15 °C.

The full-length protein as well as the shortened variant were purified using Ni-metal affinity chromatography (Ni-TED, Macherey-Nagel) followed by size exclusion chromatography (HiLoad 16/60 Superdex 200 prep grade, GE Healthcare) in either 20 mM Tris-HCl pH 8.0 or 20 mM CHES-NaOH pH 9.5, 150 mM KCl and 1 mM TCEP. The samples were concentrated via Vivaspin filtration units (Sartorius) and the concentration was spectrophotometrically determined based on their theoretical molar absorption coefficients of 28,420 M^−1^ cm^−1^ (p34ct) and 26,930 M^−1^ cm^−1^ (p34ct 1–277), respectively. After flash freezing in liquid nitrogen, the protein was stored at −80 °C.

### Crystallization

Full-length p34ct was crystallized using 0.5 – 2.0 µl of the protein solution in 20 mM Tris-HCl pH 8.0, 150 mM KCl and 1 mM TCEP, at a concentration of 10 – 12 mg/ml, added to 0.5 µl of reservoir solution. The mixture was equilibrated against the reservoir solution consisting of 100 mM Tris-HCl pH 8.0 and varying amounts of 18 – 32% PEG 550 MME, depending on the volume of protein solution per volume of reservoir in the mixture. Crystals of p34ct usually grew within 3 days at 20 °C to sizes of more than 50 × 200 × 200 µm^3^.

For data collection the crystals were flash frozen in liquid nitrogen either directly in their mother liquor or by using paraffin oil as a cryo protectant. In order to overcome the phase ambiguity some crystals were soaked in a 1.0 µl drop of 23% PEG MME 550, 100 mM Tris-HCl pH 8.0 and 100 mM KI for 1 – 5 min prior to flash freezing.

Crystals of p34ct 1–277 were grown directly from 2.0 – 4.0 µl of protein solution in 20 mM Tris-HCl pH 8.0, 150 mM KCl and 1 mM TCEP at a concentration of 10 – 12 mg/ml. The solution was placed over a reservoir containing the protein buffer including 50 – 500 mM NaCl to establish an osmotic gradient of variable strength. Crystals grew over night and reached sizes of 100 – 200 µm in all three dimensions within 3 – 5 days.

For data collection the p34ct 1–277 crystals were washed in 1.0 µl protein buffer and prepared for flash freezing by adding twice 1.0 µl of a cryo solution containing 10 mM Tris-HCl pH 8.0, 75 mM KCl, 12.5% di-ethylene-glycol, 6.25% ethylene-glycol, 6.25% MPD, 6.25% 1,2-propanediol and 6.25% glycerol to the drop and then transferring them to 1.0 µl of the same cryo solution.

### Data Collection and Structure Solution

Data collection of flash frozen crystals was performed at 100 K at beamlines ID 23.2 (ESRF) for the p34ct native data, BL 14.1 (BESSY) for p34ct anomalous KI data and ID 29 (ESRF) for p34ct 1–277 native data at wavelengths of 0.87 Å, 1.60 Å and 0.92 Å, respectively.

Both full-length p34ct and p34ct 1–277 crystallized in the cubic space group F4_1_32 with unit cell parameters ranging from a  =  b  =  c  =  257.1 Å to a  =  b  =  c  =  257.3 Å. All data sets were indexed and processed with either iMOSFLM and SCALA [22], [23] or XDS [24]. *De novo* structure solution was achieved using SHELX C/D/E [25] employing a SIRAS approach by combining anomalous p34ct KI data at 4.2 Å with a highly isomorphous p34ct native data set at 3.8 Å. Initial electron density maps allowed to manually build a preliminary p34ct model using the programs O and COOT [26], [27] and comprised the N-terminal domain of the protein. Model building was supported by results from secondary structure predictions utilizing the Phyre^2^ algorithm [28].

The preliminary model was used to solve the higher resolution p34ct 1–277 structure by molecular replacement via Phaser [29]. The model of the p34ct N-terminal domain was adjusted and extended in COOT [27] and refined to a resolution of 2.8 Å using Phenix [30]. All Figures containing structures were generated using PyMOL [31].

### Multi Angle Light Scattering

To determine the molecular mass of p34ct and p34ct 1–277 in solution, multi angle light scattering measurements (MALS, DAWN HELEOS II, Wyatt Technology) combined with refractive index detection (Optilab t-rEX, Wyatt Technology) were performed [32], [33]. The samples were diluted to a concentration of 30 µM in 20 mM CHES-NaOH pH 9.5, 150 mM KCl and 1 mM TCEP and loaded onto a Superdex 200 10/300 GL analytical size exclusion chromatography column (GE Healthcare), which was coupled to the MALS detector. The flow rate was set to 0.5 ml/min with an injection volume of 100 µl and the light scattering signal was collected at 293 K.

### DNA Binding Assays

DNA binding of p34ct was investigated using native agarose gels in 25 mM Tris-HCl pH 8.5 and 19.2 mM glycine. To prepare dsDNA substrates two 50-mer oligonucleotides (NDT, GACTACGTACTGTTACGGCTCCATCTCTACCGCAATCAGGCCAGATCTGC and NDB, GCAGATCTGGCCTGATTGCGGTAGAGATGGAGCCGTAACAGTACGTAGTC) were annealed by incubating the mixture in 100 mM KCl at 85 °C for 10 min followed by slowly cooling the samples to room temperature. For interaction studies 1 – 2 µM of ssDNA (NDB) or dsDNA (NDT/NDB) were used with 10 – 20 µM protein in a total reaction volume of 10 µl. The samples were incubated for 20 – 30 min at 4 °C and then supplemented by 10 µl loading dye, followed by an additional incubation for 10 min at 4 °C. Finally 20 µl of each sample were loaded onto 0.8% agarose gels, which were run at 50 V and 4 °C for 4 – 6 hours. Midori Green (Biozym Scientific) was used to visualize the DNA. As positive control for ssDNA and dsDNA binding we used DNA Polymerase I from *Bacillus caldotenax*.

## Results and Discussion

### Crystallization and Structure Solution of p34ct and p34ct 1–277

Crystals of p34ct grew in the highly symmetric space group F4_1_32 and diffracted to a resolution of 3.8 Å. To solve the phase problem we initially tried to use the intrinsic zinc signal of the protein's C-terminal zinc-binding domain. However, despite the clear presence of zinc as inferred from the absorption signal at the zinc edge (not shown), this approach failed due to the low anomalous signal intensities. We finally succeeded in achieving a *de novo* solution using derivative data of a potassium iodide soaked crystal at 4.2 Å.

The initial low resolution electron density map revealed the presence of approximately half of the protein, with one molecule in the asymmetric unit. Based on secondary structure prediction results using the Phyre^2^ algorithm [28] it was clear that p34ct contains an N-terminal domain and an additional C-terminal domain including a C4 zinc-finger motif, the latter of which could not be located in the electron density maps. Analysis of the p34ct crystals by mass spectrometry, however, indicated that the crystals were composed predominantly of the full-length protein (data not shown). This led us to conclude, that the p34ct C-terminal domain is disordered within the crystal lattice and likely not involved in any crystal contacts, which is in line with the fact that zinc was present in the crystal, but did not give rise to a significant anomalous signal. Using Cα-traces of structural homologues, suggested by the Phyre^2^ analysis [28], we were able to manually built a preliminary model of p34ct's N-terminal domain at 3.8 Å resolution. The anomalous derivative data used for phasing revealed a total of 8 iodide ions within the electron density map, bound to the N-terminal domain of the protein (Figure S1). The iodide ions were found at six different sites, with 4 out of the 8 ions being present in closely spaced pairs, showing a distance of 5 – 7 Å between the single ions (Figure S1, D and E).

Based on the knowledge gained through the preliminary p34ct structure, we designed a construct comprising residues 1 to 277 of p34ct, corresponding to its N-terminal domain and thus removing the flexible and unstructured part of the protein. The p34ct 1–277 variant also crystallized in space group F4_1_32 with almost exactly the same unit cell dimensions of a  =  b  =  c  =  257.1 Å (Table 1). However, diffraction was significantly improved and a highly redundant data set to a resolution of 2.8 Å could be obtained. The structure of the p34ct 1–277 N-terminal domain was solved by molecular replacement with the truncated preliminary p34ct model serving as template for phasing.

**Table 1:** Data Collection and Refinement Statistics.

Data Collection	p34ct	p34ct KI	p34ct 1–277
Space Group	F 4_1_ 3 2	F 4_1_ 3 2	F 4_1_ 3 2
Cell Dimensions			
a, b, c [Å]	257.31, 257.31, 257.31	257.07, 257.07, 257.07	257.11, 257.11, 257.11
α, β, γ [°]	90, 90, 90	90, 90, 90	90, 90, 90
Resolution [Å]	3.8	4.2	2.8
Wavelength [Å]	0.8726	1.6000	0.9199
Unique Reflections	7,694	10,090	18,512
I/σI	16.2 (1.4)	18.7 (2.1)	20.9 (2.9)
Rmerge [%]	14.2 (142.1)	14.5 (78.8)	15.3 (259.8)
Rpim [%]			1.9 (29.7)
Completeness [%]	99.8 (100.0)	99.9 (100.0)	100.0 (100.0)
Redundancy	10.1	46.4	70.5
SigAno		1.288	

Refinement	p34ct	p34ct KI	p34ct 1–277
Resolution			2.8
Number of Atoms			3,190
R_work_ (R_free_)			21.9 (23.8)
Mean B-Factor [Å^2^]			96.5
R.m.s. Deviations			
Bond Lengths [Å]			0.004
Bond Angles [°]			0.783
Ramachandran Statistics			98.5/1.5/0.0/0.0

Values in parentheses refer to the highest resolution shell. Rmerge  =  ∑_hkl_ ∑_i_ (|I_i_(hkl) − <I(hkl)>|)/∑_hkl_ ∑_i_ I_i_(hkl) and Rpim  =  ∑_hkl_ (√(1/(N-1)) ∑_i_ (|I_i_(hkl) − <I(hkl)>|)/∑_hkl_ ∑_i_ I(hkl) where I is the measured intensity and <I> is the averaged intensity of each unique reflection with indices hkl. I/σI indicates the average of the intensity divided by its average standard deviation. R_work_  =  ∑_hkl_ ||F_o_| − |F_c_||/∑_hkl_ |F_o_| where F_o_ and F_c_ are the observed and calculated structure factors, respectively. R_free_ is the same as R_work_, calculated for the 5% of the data that was randomly omitted from refinement. The Ramachandran statistics indicate the fraction of residues in the most favored, allowed and disallowed regions of the Ramachandran plot, respectively, in addition to the fraction of rotamer outliers observed.

**Figure 1:**
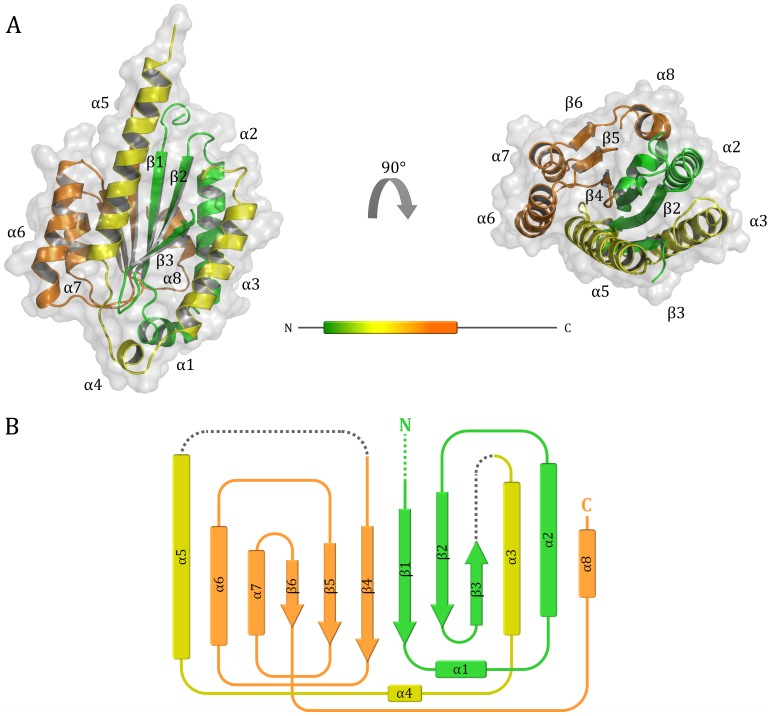
Overall structure of p34ct and analysis of the MIDAS region. (A) Front view of the p34ct 1–277 vWA domain (left) and view from the top (right) with the color coding corresponding to the N-terminal (green), central (yellow) and C-terminal (orange) part of the domain, respectively. The location of the vWA domain within full-length p34ct is indicated in the equally colored bar representation. (B) The topology diagram of the vWA fold shows that its architecture can be distinguished from a typical Rossmann fold by β-strand β3, which is anti-parallel in all vWA domains.

**Figure 2:**
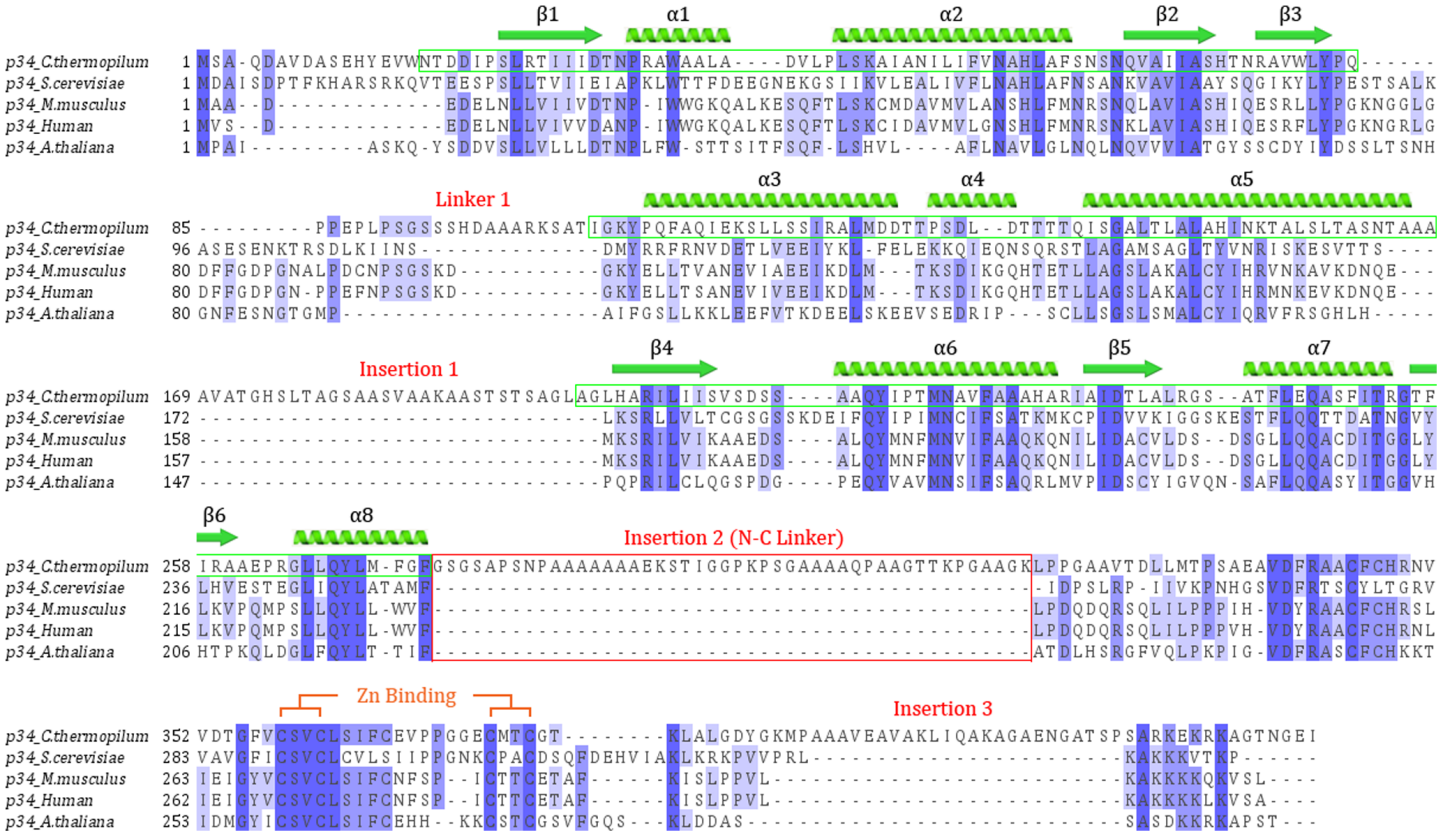
Multiple sequence alignment of p34 proteins from five different organisms. The alignment was obtained with MUSCLE [56] and visualized via JalView [57] after manual modification. Regions visible in the p34ct vWA structure are boxed in green and secondary structure elements are indicated above the sequence, with arrows representing β-strands while coils are used for α-helices. Conserved residues between species are colored in different shades of blue, depending on the degree of conservation. The N-C-Linker present in *C. thermophilum*, but absent in *S. cerevisiae*, *M. musculus*, human and *A. thaliana* p34, is highlighted in red. The highly conserved C-terminal C4 zinc finger motif is indicated in orange.

Overall, 17 amino acids at the N-terminus of p34ct 1-277, in addition to the hexa-histidine tag, as well as 2 long linker regions (residues 86 to 105 and 169 to 197) were not accounted for in the electron density map (Figures 1 and 2). The final model thus contains 209 out of the 277 residues and was refined to R_work_ and R_free_ values of 0.2187 and 0.2380, respectively, with good overall stereochemistry (Figure S2). 98.5% of all the residues are located in favored regions in the Ramachandran plot and no outliers are observed (Table 1).

### Structure of p34ct 1–277

The N-terminal domain of p34ct consists of a central 6-stranded β-sheet with 5 parallel (β1, β2, β4, β5 and β6) and one shorter anti-parallel β-strand (β3), which is surrounded by a total of 3 α-helices on either side (α2, α3, α8 on one side and α5, α6, α7 on the other). Two additional, much shorter α-helices are located at the C-terminal end of the central β-sheet of the domain (α1 and α4, Figure 1). Surprisingly, the overall fold reveals a von *Willebrand Factor A* (vWA) like architecture (Figure 1B), with high structural similarity to the A1 domain of the *von Willebrand Factor* protein, a blood glycoprotein involved in hemostasis and platelet aggregation [34]. A vWA like domain has previously not been reported for p34. In line with this result our searches using the primary sequence of both human and *C. thermophilum* p34 did not return a vWA like feature via BLAST and the SMART domain annotation database, respectively [35], [36]. However, a Phyre^2^ secondary structure analysis did predict the vWA like fold and suggested several vWA containing structural homologues to p34 [28]. Interestingly, also the N-terminal half of p44, the binding partner of p34 within TFIIH, is predicted to contain a vWA like domain.

While the XPD, XPB and p44 subunits of TFIIH are relatively well conserved between different organisms, with mean residue identities of 52%, 50% and 35%, respectively, there seems to be higher variability in p34 [13]. Here, the mean sequence identity is reduced to 30% within a set of 63 different species [13] and reaches 28% when comparing the human to *C. thermophilum* p34. Interestingly, the *C. thermophilum* p34 is also larger than its human orthologue, with extensions at both N- and C-termini and flexible linker insertions. In total the size of p34 increases by 39% and reveals the presence of one variable linker and three linker insertions when compared to its human, mouse, yeast and plant homologues (Figure 2). The variable linker is located in a region connecting β-strand β3 to helix α3, which is disordered in our crystal structure (Figure 2, Linker 1). The three insertions encompass residues 166 – 199, 275 – 320 and 391 – 414 and are not present in the other species. The first of these is located in the vWA domain (Figure 2, Insertion 1). It connects helix α5 back to the β-sheet core and is again disordered in the p34ct structure. The second insertion bridges the N-terminal vWA domain and the highly conserved C-terminal region (Figure 2, Insertion 2). It spans more than 40 residues and most likely introduces high flexibility between the N-terminal and C-terminal parts of the protein. The third insertion is located at the far C-terminus beyond the C4 zinc-binding motif (Figure 2, Insertion 3).

### The p34ct 1–277 Structure in Light of other vWA Domains

With the N-terminal domain of p34ct assuming a vWA like fold it was not surprising that a closer investigation using the DALI server yielded 140 similar structures with a Z-score higher than 10.0 and r.m.s.d. values between 2.1 and 3.3 Å [37]. Most of the molecules with the highest scores either correspond to the name giving human *von Willebrand Factor (vWF) A1* domain itself [38] or complexes of this domain from mouse or human origin with a variety of different proteins [39]–[42].

**Figure 3:**
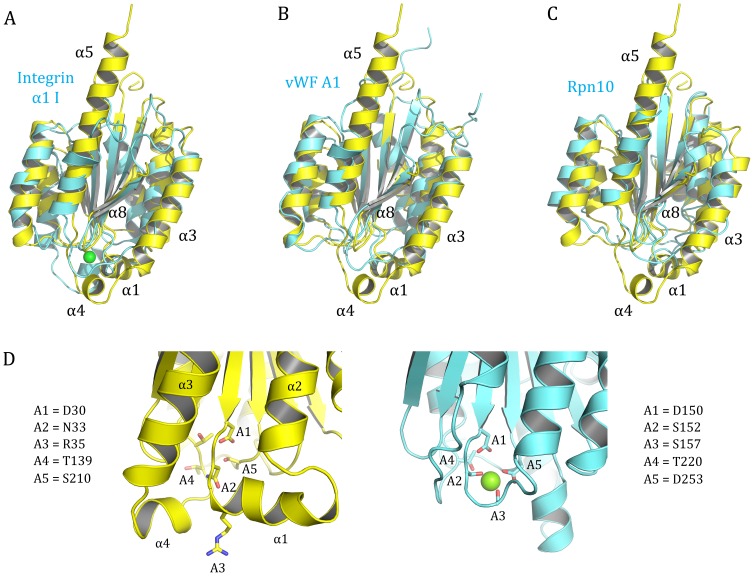
Structural similarity of p34ct compared to other vWA domains. Superposition of the p34ct 1-277 structure (yellow) with (A) the human Integrin α1 I domain (pdb entry 1PT6), (B) the human vWF A1 domain (pdb entry 1AUQ) and (C) Rpn10 from *Schizosaccharomyces pombe* (pdb entry 2×5N, each in cyan). The putative metal coordination site in p34ct is enlarged (D) and compared to the MIDAS motif in human Integrin α1 (cyan), with the 5 MIDAS elements (A1 – A5) shown in stick representation and the bound Mg^2+^ ion in Integrin α1 (1PT6) depicted as a green sphere. Residues corresponding to A1 – A5 are depicted next to the structural models.

Superposition of the p34ct 1–277 structure with proteins suggested by the DALI search, revealed that the overall fold is highly conserved, even among rather distant homologues involved in different cellular pathways, such as the I-domain of the cell adhesion molecule Integrin α1 [43], the *von Willebrand Factor* A1 domain [38] and the 26S proteasome regulatory subunit Rpn10 [44], which all share the central 6-stranded β-sheet core, surrounded by 6 α -helices (Figures 3A, 3B and 3C, respectively). However, there are also some remarkable structural features in p34ct 1–277 that have not been observed in the vWA domains of most other proteins. Helix α3 is significantly longer in p34ct 1–277, while helix α8 is relatively short and often found to be longer in other vWA containing proteins, except for Rpn10 and the vWA domain of the DNA dependent helicase Ku70 [45]. Furthermore, the two helices at the C-terminal end of the central β-sheet (α1 and α4, Figure 3) are often replaced by short loops in other vWA folds. The most striking feature, however, is helix α5, which is about twice as long compared to the corresponding helix in other vWA proteins, considerably protruding outward from the top side of the domain (Figure 3 A–C). The drastic extension of this helix might also explain the presence of the rather long linker region required to provide the connection back to the β-sheet core, as observed in p34ct 1–277 (Figure 2, Insertion 1). However, as this linker is absent in p34 sequences of other organisms, it remains unclear, if helix α5 is similarly prominent in these species. Based on our p34ct vWA structure, multiple sequence alignments and secondary structure predictions, we propose helix α5 to be at least one or two turns shorter in human and mouse p34 and the p34 homologue Tfb4 in *S. cerevisiae*, respectively. Another possibility would be a shift of helix α5 more to the center of the domain to permit the connection to β4 (Figure 2).

While no vWA domain is known to bind nucleotides, many of them are capable of coordinating metal ions via a noncontiguous sequence motif termed metal-ion-dependent adhesion site (MIDAS). For example, all vWA domains in integrin α subunits and most integrin β representatives contain a perfectly conserved MIDAS motif, which is formed by 3 loops at the C-terminal end of the central β-sheet of the vWA fold [46]. The MIDAS motif consists of 5 residues in the order **D**-x-**S**-x-**S** … **T** … **D**, which are in the following referred to as A1 to A5. In p34 from *C. thermophilum* (ct), homo sapiens (h) and several other species the aspartic acid at position A1 is perfectly conserved. However, in both p34ct and p34h the MIDAS motif is disrupted by a helical insertion (α1) directly after A1, which is often rich in one or two tryptophans, leading to non-conservative substitutions in A2 and A3 (Figure 3D). While A4 seems to be conserved, the aspartic acid at position A5 is replaced by a serine in p34. Hence, p34 is not likely to bind a metal ion via the MIDAS motif, which is consistent with our p34ct vWA structure where no bound ligands were observed.

### Oligomeric State and DNA Binding Properties of p34ct in Solution

**Figure 4:**
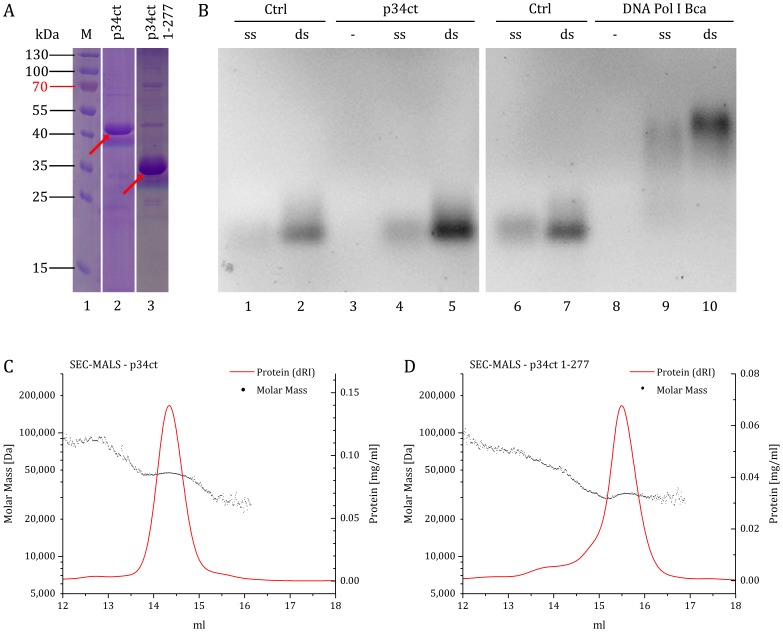
DNA binding properties and oligomeric state of p34ct samples. SDS-PAGE of purified samples after size-exclusion chromatography, with the position of each p34ct construct indicated by red arrows (A). For DNA binding assays (B) the samples were separated on native agarose gels and the DNA visualized via Midori Green staining. Neither p34ct (B, left) nor p34ct 1–277 (not shown) were able to bind single stranded (ss) or double stranded (ds) DNA. DNA Polymerase I from *Bacillus caldotenax* served as a positive control for DNA binding (B, right). The multi angle light scattering analysis of p34ct (C) and p34ct 1–277 (D) samples was coupled to size-exclusion chromatography. The sample concentration in mg/ml as a function of the differential refractive index (dRI) is shown in red whereas the calculated molar mass is indicated as a scatter plot, with one data point per measurement and second (C and D).

Although many studies, including Cryo-EM data [47], [48], suggest the 7-subunit core of the general transcription factor TFIIH to consist of single subunits, the true oligomeric state of each subunit within TFIIH has not been resolved. Recently, Kainov *et al.* reported dimeric forms of both Tfb2 (p52) and Tfb5 (p8) that undergo a dimer to hetero-tetramer transition upon interaction with each other [16], [49]. As many proteins are known to self-associate in the absence of their respective binding partners [50], we analyzed the oligomeric state of full-length p34ct and p34ct 1–277 in solution using multi angle light scattering coupled to size-exclusion chromatography. The obtained data yielded molecular weights of 47 kDa and 32 kDa, respectively, closely matching the predicted values of 46,767 Da for p34ct and 31,941 Da in the case of p34ct 1–277 (Figure 4C and 4D). Hence, under the conditions tested, p34ct is monomeric in solution. In addition, an analysis of crystal packing interactions using the PDBePISA service [51] revealed no significant dimerization sites, which is in support of our solution data.

Given the DNA oriented role of the TFIIH complex in transcription and DNA repair, we also investigated the DNA binding capability of both p34 variants in solution. In case of the yeast homologue of p34, Tfb4, nucleic acid binding via a basic Lys/Arg-rich C-terminal region has recently been suggested [49]. Our data, however, could not confirm this observation since neither p34ct 1-277 (data not shown) nor the p34ct full-length protein were able to bind to single stranded or double stranded 50-mer DNA substrates (Figure 4B). However, it cannot be ruled out that this differs among species. In p34ct the Lys/Arg-rich stretch at the remote C-terminus is disrupted by an insertion and is not as basic as in p34/Tfb4 from *S. cerevisiae* (Figure 2).

### Elucidation of Putative p44 Binding Sites

**Figure 5:**
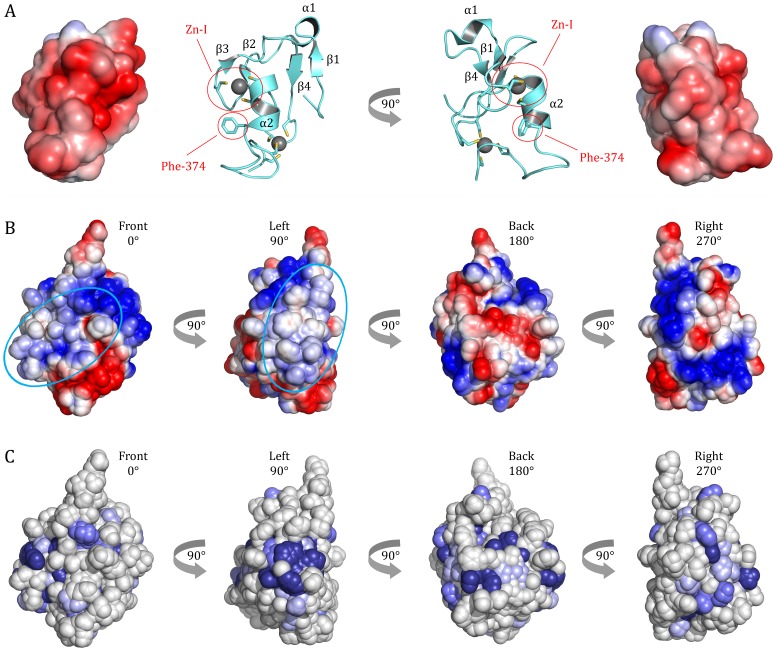
Potential binding sites for the TFIIH p44 subunit. (A) A structure of the human C-terminal domain of p44 (1Z60) is shown as a cartoon model and with the electrostatic potential (contoured at ± 3.0 k_b_T/e_c_) mapped onto its surface. The zinc ions bound to the C4C4 motif in p44 are shown in grey and the two regions most likely involved in p34 binding are circled in red. (B) The electrostatic potential contoured at ± 3.0 k_b_T/e_c_ is mapped onto the p34ct surface (A) in blue (positive), red (negative) and white (neutral) and suggests two putative binding sites for the p44ct C4C4 domain (circled in cyan). (C) The conservation of surface residues in the p34ct vWA domain is depicted, with the different shades of blue reflecting the variable degree of conservation, in analogy to the color code used in Figure 2.

With p34 being the natural binding partner of the p44 subunit in TFIIH we investigated how the interaction of both proteins could be envisioned. From previous studies it is known that residues 1–242 of human p34 and 321–395 of human p44 (corresponding to residues 1–285 and 375–534 in *C. thermophilum*, respectively) are sufficient for the formation of a tight p34–p44 complex [19]. In addition, an NMR structure of the C-terminal domain of human p44 revealed the fold of its C4C4 RING domain [20] (Figure 5A). The authors also investigated, which parts of the domain are important for interaction with p34 and observed, that the conservation of the first zinc-binding site is required for the formation of a stable complex. A single coordinating cysteine residue mutated at that position seemed to disrupt the local fold of the domain and abolished binding [20]. In addition, Phe-374, located in helix α2 close to the first zinc-binding site, seems to be important for the interaction with p34 since its mutation to a serine disrupts complex formation (Figure 5A). Overall, the authors suggested, that the interaction is more likely to be of hydrophobic than electrostatic character [20]. Interestingly, however, the overall surface charge of the p44h C4C4 domain is highly acidic, with a total of 10 aspartic and glutamic acid residues, all of which are solvent exposed (Figure 5A), and only 2 positively charged residues (Arg and Lys).

With most of the residues around the first zinc-binding site and especially helix α2, including the phenylalanine, being highly conserved in p44, we thus propose that these two sites contact p34 at a mainly hydrophobic and potentially slightly basic region. Two predominantly hydrophobic patches can be found on the front and left side of the p34 vWA domain (β3, α5, α6, α7, Figure 1A and 5B). Binding of p44ct C4C4 to the back of the vWA domain (β6, α8) is unlikely, as there is a stretch of solvent exposed, highly negatively charged residues. Similarly we exclude binding to the right face of the domain (α2, α3), as most residues in helix α3 and the hydrophobic part of α2 are not very well conserved (Figures 2 and 5B–C). Hence, the most likely interface is found at the front and/or left side of the vWA domain (β3, α5, α6, α7), with most of the surface exposed residues in helices α6 and α7 being strongly hydrophobic and highly conserved (Figures 2 and 5B–C). Along that line also the lower portion of helix α5 shows a high degree of conservation and provides a slightly positive contribution to the overall surface potential, which could favorably accommodate part of the negative contribution by p44 C4C4. Taken together, these considerations make the front and left side of the vWA domain (β3, α5, α6, α7) the most attractive target for binding of the p44 C4C4 domain (Figures 1 and 5B–C).

### Implications for Protein-Protein Interactions within TFIIH

The typical vWA domain is known for its capability to mediate protein-protein interactions. It is thus tempting to speculate how both p44 and p34 function within the context of the general transcription factor TFIIH. It has been shown that the p44 vWA domain contacts a C-terminal region in XPD, thereby stimulating its helicase activity [15]. In turn p34 vWA binds strongly to the C-terminal C4C4 RING domain of p44 [19], which is essential for transcriptional activity [52], potentially forming a regulatory triad with XPD and p44. However, only very little is known about the function of p44's central and p34's C-terminal zinc finger domain, respectively. Based on the knowledge that vWA domains are capable to assume multiple protein-protein interfaces [41], [42], [53], [54] it is very likely that the vWA domains of both p44 and p34 are involved in multiple contacts as well, presumably providing stability and serving as a platform and mediator for other subunits, while their zinc-finger motifs could enable them to perform additional roles, which are so far unknown.

Recently, it was shown that a stable 5-subunit minimal complex of Rad3, Tfb1, Tfb2, Tfb4 and Ssl1 (corresponding to the human XPD, p62, p52, p34 and p44 proteins) can only be obtained, when Tfb4 (p34) is included during co-expression [55]. If Tfb4 was omitted, the complex lacked Tfb2 (p52), which suggests that Tfb4 (p34) is required for integration of Tfb2 (p52) into the minimal core complex in yeast. This data supports the notion that p34 is involved in multiple protein-protein interactions within TFIIH. Further analysis will be required to decipher this network of interplay and regulation between TFIIH subunits, especially in the context of transcription and nucleotide excision repair, to gain insight into the molecular requirements for a fully functional TFIIH complex.

### Accession Numbers

The data have been deposited and the PDB Code 4PN7 has been assigned.

## Supporting Information

Figure S1 **Environment of the iodide ions used for SIRAS phasing.** The local environment of the 8 iodide ions used for SIRAS phasing is depicted in A – F, with the anomalous electron density for each iodide ion contoured at 5.0 sigma (orange mesh). The iodide ions are depicted as grey spheres, while the coordinating residues at each site are shown in stick representation. Secondary structure elements of symmetry related molecules are indicated by a prime symbol following the α or β labeling, respectively.
(TIF)

Figure S2 **Final model and 2F_o_–F_c_ electron density map of the p34ct structure.** The final model of p34ct is shown in stick representation for a portion of helix α5 (A) and the α7β6 transition (B) with the 2F_o_–F_c_ electron density map contoured at 1.0 sigma.
(TIF)
