# Supplementary figures and images for: The Structure of the TFIIH p34 Subunit Reveals a Von Willebrand Factor A Like Fold
Source: PLoS One. 2014 Jul 11;9(7):e102389. doi: 10.1371/journal.pone.0102389 (PMC4094531; doi:10.1371/journal.pone.0102389)

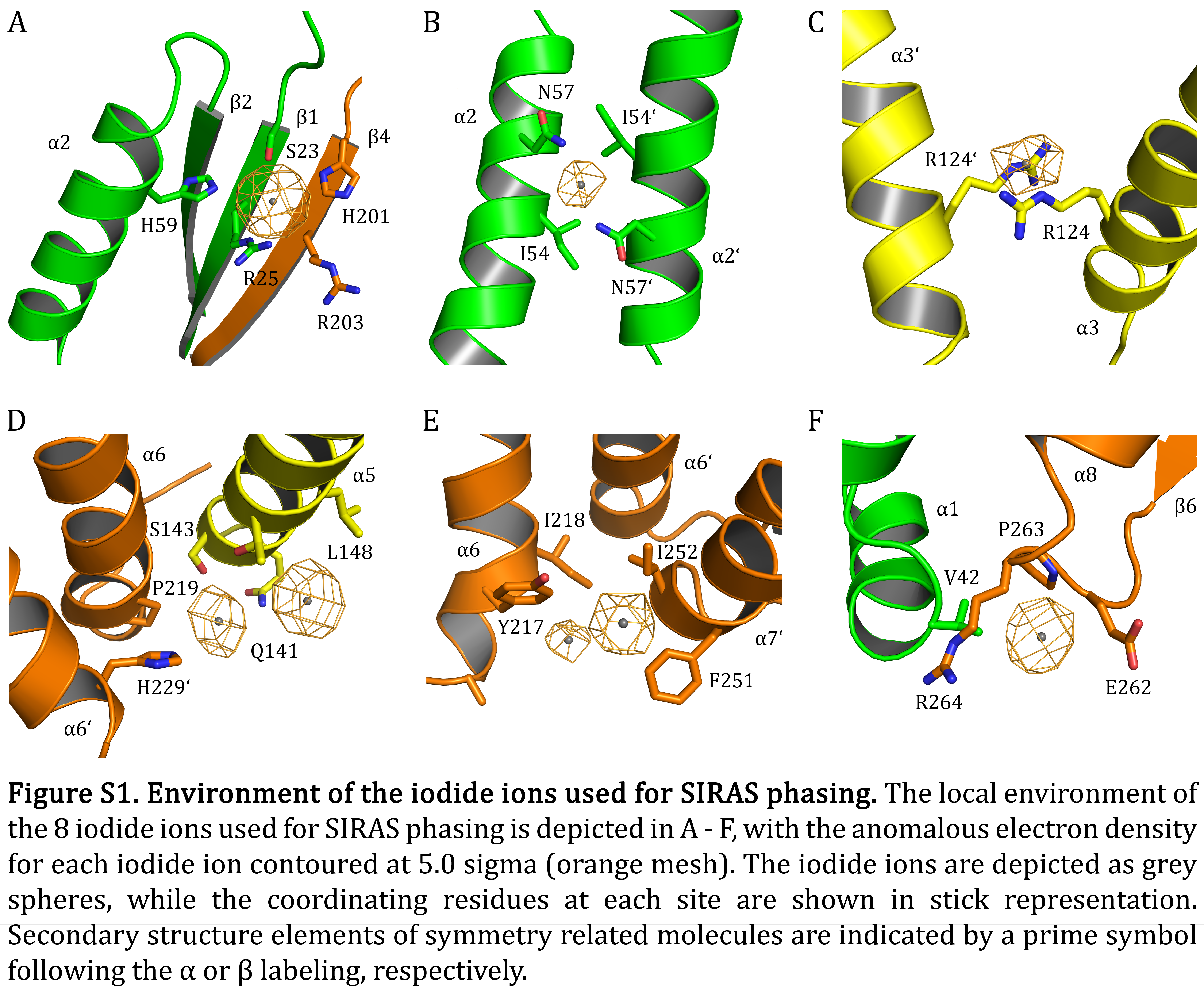

Supplement: Figure S1 — Environment of the iodide ions used for SIRAS phasing. The local environment of the 8 iodide ions used for SIRAS phasing is depicted in A – F, with the anomalous electron density for each iodide ion contoured at 5.0 sigma (orange mesh). The iodide ions are depicted as grey spheres, while the coordinating residues at each site are shown in stick representation. Secondary structure elements of symmetry related molecules are indicated by a prime symbol following the α or β labeling, respectively. (TIF) [file pone.0102389.s001.tif]

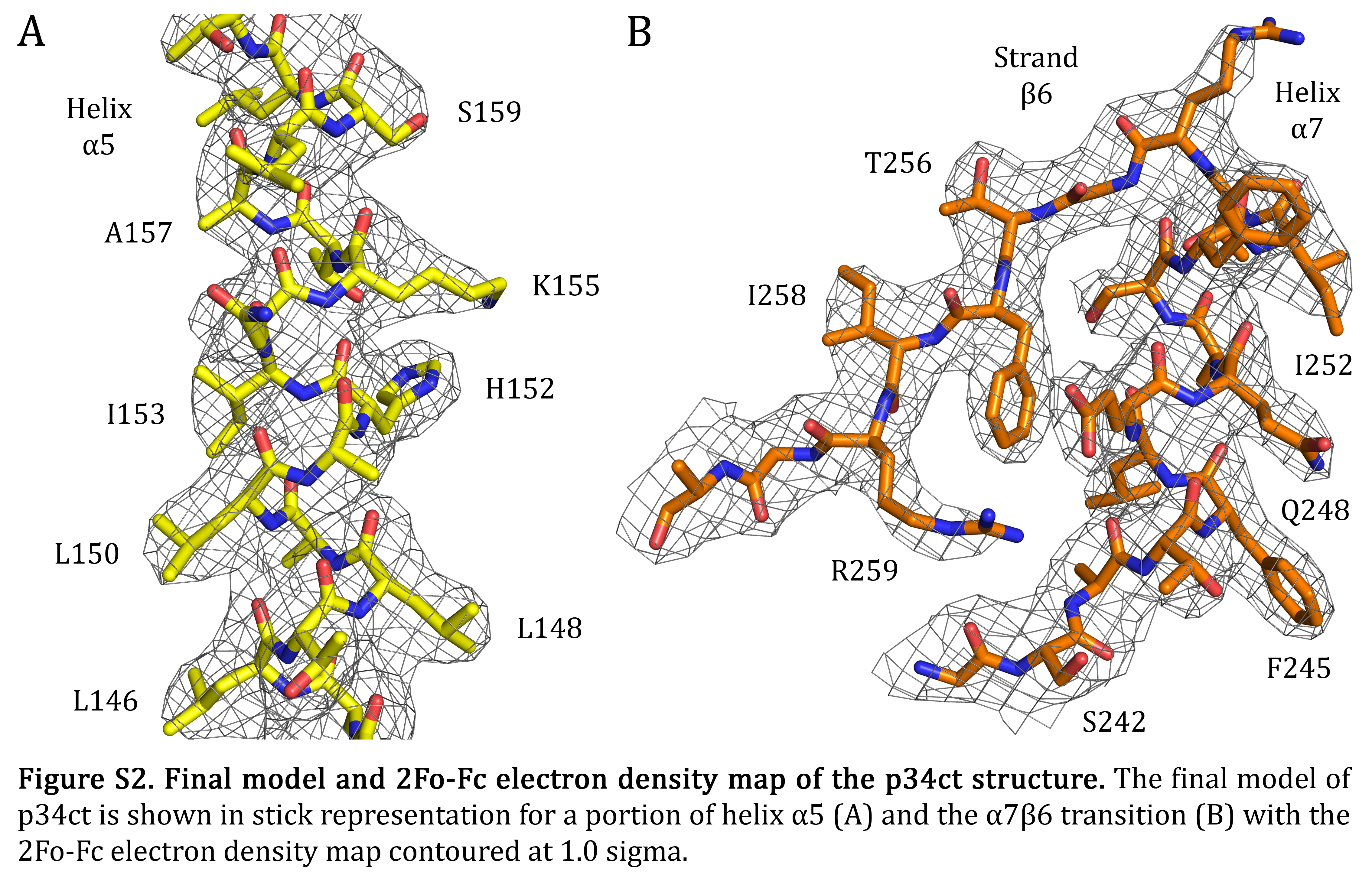

Supplement: Figure S2 — Final model and 2Fo–Fc electron density map of the p34ct structure. The final model of p34ct is shown in stick representation for a portion of helix α5 (A) and the α7β6 transition (B) with the 2Fo–Fc electron density map contoured at 1.0 sigma. (TIF) [file pone.0102389.s002.tif]
